# Investigation into the Effect of Acoustic Radiation Force and Acoustic Streaming on Particle Patterning in Acoustic Standing Wave Fields

**DOI:** 10.3390/s17071664

**Published:** 2017-07-19

**Authors:** Shilei Liu, Yanye Yang, Zhengyang Ni, Xiasheng Guo, Linjiao Luo, Juan Tu, Dong Zhang, Jie Zhang

**Affiliations:** 1 Key Laboratory of Modern Acoustics (MOE), Department of Physics, Collaborative Innovation Centre of Advanced Microstructure, Nanjing University, Nanjing 210093, China; lsl666@foxmail.com (S.L.); dz1622045@smail.nju.edu.cn (Y.Y.); 15905191508@163.com (Z.N.); linjiao@nju.edu.cn (L.L.); juantu@nju.edu.cn (J.T.); 2 The State Key Laboratory of Acoustics, Chinese Academy of Science, Beijing 100190, China; 3 Department of Mechanical Engineering, University of Bristol, Bristol BS8 1TR, UK; J.Zhang@bristol.ac.uk

**Keywords:** acoustic radiation force, acoustic streaming, particle image velocimetry, particles patterning, acoustic particles manipulation

## Abstract

Acoustic standing waves have been widely used in trapping, patterning, and manipulating particles, whereas one barrier remains: the lack of understanding of force conditions on particles which mainly include acoustic radiation force (ARF) and acoustic streaming (AS). In this paper, force conditions on micrometer size polystyrene microspheres in acoustic standing wave fields were investigated. The COMSOL^®^ Mutiphysics particle tracing module was used to numerically simulate force conditions on various particles as a function of time. The velocity of particle movement was experimentally measured using particle imaging velocimetry (PIV). Through experimental and numerical simulation, the functions of ARF and AS in trapping and patterning were analyzed. It is shown that ARF is dominant in trapping and patterning large particles while the impact of AS increases rapidly with decreasing particle size. The combination of using both ARF and AS for medium size particles can obtain different patterns with only using ARF. Findings of the present study will aid the design of acoustic-driven microfluidic devices to increase the diversity of particle patterning.

## 1. Introduction

The use of acoustic radiation force (ARF) to trap and pattern particles has been rapidly developed in recent years [1]. Particularly, using this principle, various acoustic-driven microfluidic devices have been designed and fabricated to trap and pattern particles in biomedical and chemical analysis and other research fields to observe and control cells, drugs, particles, and bubbles [2,3]. In acoustic microfluidic devices, either bulk acoustic waves (BAWs) [4,5] or surface acoustic waves (SAWs) [6,7] can be used to generate acoustic standing wave fields to trap particles. BAWs can be generated from a piezoelectric plate in a fluid volume. A number of piezoelectric plates can be used to make an array system surrounding an area of interest to generate controllable acoustic standing wave fields. Multiple studies have been performed to improve the efficacy of such devices. For example, Grinenko et al. [8] designed a water-surrounding device to significantly reduce the effect of the reflections from piezoelectric plates on the expected acoustic standing wave fields. Courtney et al. [9] and Bernassau et al. [10] investigated the effect of loading signal phases on the resultant acoustic standing wave fields from a piezoelectric plate layout with various polygonal shapes. Zhang et al. [11] combined BAWs and SAWs to realize multi-scale particle patterning.

The force conditions on a particle in acoustic standing wave fields in a compressible fluid medium mainly include ARF and acoustic streaming (AS) and they have been investigated by a number of researchers. For example, Gorkov et al. [12,13] proposed a model to calculate the ARF on a compressible spherical object in an arbitrary pressure field. Wiklund et al. [14] pointed out different types of AS in an acoustic-driven microfluidic device, including inner and outer boundary layer acoustic streaming, Eckart streaming, and cavitation microstreaming. Lei et al. [15,16] calculated micro-streaming in a microfluidic device and demonstrated a 3D Rayleigh streaming pattern. Ahmed et al. [17,18] investigated the manipulation of cells in an acoustic field using both ARF and AS. Collins et al. [19] have demonstrated how high frequency traveling waves, as well as strong acoustic streaming, are excited and used it to achieve accurate particle manipulation, while the particles are in the order of submicrometers (300–500 nm). While, in our work, low frequency standing waves are generated by a BAW device and the arrangement rules in a standing acoustics field where particles assemble at the nodal positions are researched.

The relationship of the two competing forces, ARF and AS, has been studied by Muller et al. [20], in which theory the particles in the microfluidics are subject to two acoustic forces: the ARF from the scattering of sound waves on the particles and the Stokes drag force from the induced AS were pointed out. ARF dominates over the streaming-induced drag force for the larger polymer particles. Conversely, for smaller particles, acoustic streaming, which is a well-known phenomenon in acoustics, influences the motions of particles obviously. The principle was numerically examined by Bruus [21]. Based on the theoretical analysis mentioned above, an in-device, PIV-combined experiment was designed and conducted to experimentally verify the actual performance of ARF and AS on different sizes of micro-particles. To further support experimental data, finite element software analysis was applied to explain the different roles of ARF and AS in the chamber. Both experiment and analysis were performed in 1D and 2D acoustic fields to uncover the combined effects of ARF and AS under dimensional and spatial variations. The present study not only considered the 1D situation, but also examined the compromising effect of ARF and AS in a 2D acoustic field.

It is noted that the size of acoustic-driven microfluidic devices ranges from submicrometer to millimeter. Various theories have been proposed to explain the principles in microfluidics. In addition, the microenvironment within microfluidic devices has been recognized to be rather complex, bringing great difficulty in quantitative calculation [22,23]. In general, techniques involved in measuring and manipulating particles in microfluidic fluid are more complicated than those in macroscopic fluid [24,25]. Particularly, the dynamic characteristics of the microfluidic flow, mainly ARF and AS, are more difficult to be experimentally investigated due to the diverse produce cause [26,27]. The flow field, however, can be accurately measured by particle imaging velocimetry (PIV) a technique in which velocity vectors of fluorescent tracing particles can be calculated based on recorded fluorescence patterns [28,29].

In this paper, four piezoelectric plates with a rectangular element layout in a water chamber are used to generate acoustic standing wave fields. Three types of polystyrene microspheres with various sizes are placed in the acoustic standing wave fields. PIV technique is used to scale the velocity fields around these particles. COMSOL^®^ Multiphysics software is used to simulate acoustic pressure, particle velocity distributions, and particle patterning process. The effect of ARF and AS on particle trapping and patterning is finally discussed.

## 2. Theory

When a particle is placed in an acoustic standing wave field in a compressible fluid medium, ARF is induced by the existence of non-linear terms of the vibration in the medium and it can push the particle toward force-balanced point and it is given by [18,30,31],

(1) $$F_{r}=-(\frac{\pi p_{0}{}^{2}V_{p}\beta_{m}}{2\lambda })\varphi (\beta ,\rho )\sin(2kx)$$

where, $\varphi =\frac{5\rho_{p}-2\rho_{m}}{2\rho_{p}+\rho_{m}}-\frac{\beta_{p}}{\beta_{m}}$, $p_{0}$ is the acoustic pressure, $V_{p}$ the particle volume, λ the wavelength in the fluid medium, k the wave number, x the displacement along the wave propagation direction, $\rho_{m}$ the density of water, $\rho_{p}$ the density of the particle, $\beta_{m}$ the compressibility of the fluid medium, and $\beta_{p}$ the compressibility of the particle. As long as the velocity of a particle is different from that of the surrounding medium, the particle is also being dragged by the Stokes force which is given by [18],

(2) $$F_{v}=-6\pi \eta rv$$

where η, r, and v are the medium viscosity, the radius of the particle, and the relative speed of the media between the particle, respectively. Meanwhile, the acoustic vibration can also cause different fluid motions—for example, boundary flow, internal flow, attenuation flow, and cavitation flow—and all these are related to AS [24,32].

From Equation (1), it can be seen that the ARF conforms to a sinusoidal distribution with an interval of a half wavelength in space. Driven by the surrounding force, the particle will stay at a force-balanced point which depends on the sign of φ. If φ is positive, the particle will gather at a node (pressure is zero) in the standing wave, otherwise, at an antinode (pressure is maximum) [33]. It is noted that the ARF is proportional to the cube of particle radius and the Stokes force is proportional to the particle radius [14,34]. This indicates that, for a large particle, the ARF is dominant in its movement toward to a force-balanced position. For a small particle, however, it is more likely to comply with the movement of fluid, i.e. AS.

## 3. Experimental Setup

**Figure 1:**
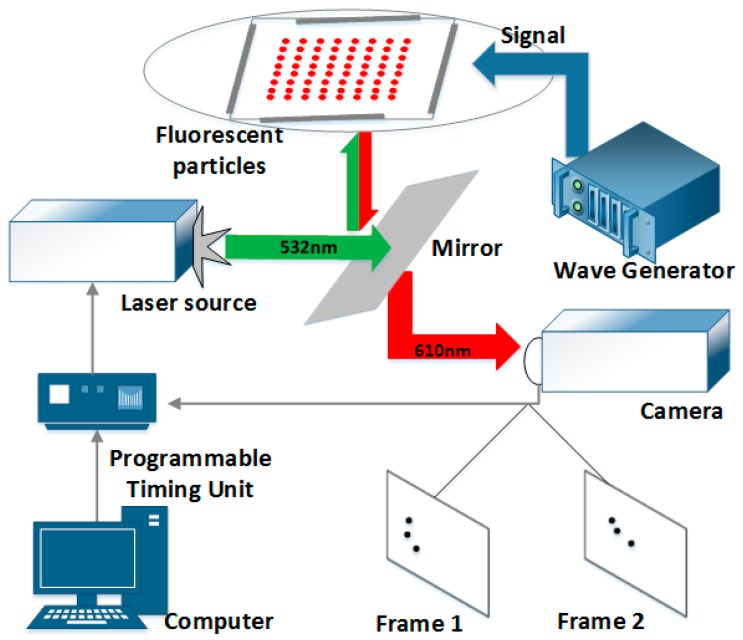
The experimental measurement system consisted of a signal electrical excitation system, a BAW microfluidic device, and a PIV system.

Figure 1 shows the PIV system used to measure the fluid velocity in an acoustical standing wave field. In the system, a BAW microfluidic device is used to generate acoustical standing wave fields, the configuration of which has been explicitly demonstrated by Drinkwater in Figure 1b in reference [1]. The BAW device is made up of a water-immersive rectangular-shape chamber with a size of 20 mm×20 mm and four attached piezoelectric plates with a size of 1 mm×2 mm× 15 mm and a resonance frequency of 6.9 MHz. A wave generator (Agilent 33250A, Santa Clara, CA, USA) is connected to the piezoelectric plates and provides sinusoidal wave signals of 6.9 MHz. In the PIV system, an inverted microscope (Olympus IX71, Japan) is used for imaging. A double pulse laser generator (EKSPLA-NL303D-10, Lithuania, wavelength = 532 nm) is used for illumination and the fluorescent pattern is recorded by a high resolution camera (Lavision Imager Pro SX, Göttingen, Germany). The cross-correlation PIV software DaVis 8.0 (Lavision, Göttingen, Germany) is used to calculate the velocities of particles from the measured images. The time synchronization unit of the whole system is the PTU time synchronizer (Lavision, Göttingen, Germany), which controls the correlation coefficient of the exposure parameter.

Polystyrene fluorescent microspheres with various sizes were added into the water chamber for the measurements. These particles are, Thermo Fisher^®^ R0100 (Thermo Fisher, Fremont, CA, USA) with *d* = 1 μm and *ρ_p_* = 1.05 g/cm^3^, Thermo Fisher^®^ R0300 (Thermo Fisher, Fremont, CA, USA) with *d* = 3 μm and *ρ_p_* = 1.05 g/cm^3^ and Spherotech^®^ 73-FP-10056 (Spherotech, Lake Forest, IL, USA) with *d* = 10 μm and *ρ_p_* = 1.05 g/cm^3^. Note that all these parameters lead to positive φ and this means that ARF should push all particles toward the nodes of the standing waves.

In the experimental measurements, the fluorescent microspheres in water were exposed under double-pulsed lasers within a very short interval time (100 μs) and the captured position information of tracer particles was transferred to the computer [35]. The local displacement vector of the tracer particles between the first and second illumination was determined for each interrogation area by means of cross-correlation calculation under the assumption that all particles within one interrogation area moved homogeneously between two illuminations. The flow field was finally obtained by the delay time between two pulses and the magnification of imaging. During this procedure in the experiment, each image was divided into small interrogation areas, whose sizes were 96 × 96 pixels with 75% overlapping. All the velocity filed is calculated by the DaVis 8.0. The time interval between two frames being considerably short, the PIV result could be regarded as the instantaneous velocity of particle motion [36].

## 4. Simulation and Experimental Data Analysis

It is noted that the PIV-measured velocity fields denote all motions of the particles, which is the combination of the ARF and the AS. In this paper, all piezoelectric plates in the BAW microfluidic device were operated at the same phase, the particle patterning was performed in both one-dimensional and two-dimensional standing wave fields.

In the simulations, COMSOL^®^ Multiphysics 5.2a (COMSOL, Burlington, MA, USA) was used to explore the characteristics of acoustic radiation fields and microstreaming in the microfluidic device. To save the memory of calculating, “Pressure Acoustic, Frequency Domain” physics in COMSOL^®^ (COMSOL, Burlington, MA, USA) was utilized to simulate 2λ×2λ-sized first-order acoustic fields, which takes the form

(3) $$\nabla \cdot \left(-\frac{1}{\rho_{0}}\nabla p\right)-\frac{\omega^{2}}{\rho_{0}c^{2}}p=0$$

where ω is the angular frequency (2π×6.9 MHz in this simulation), c the sound speed, $\rho_{0}$ the density, and p the pressure. The boundaries of the simulation area were considered normal displacement boundary conditions (Amplitude: 5 nm). COMSOL^®^’s “Laminar Flow” physics was used to calculate the second-order acoustic streaming fields. The mechanic force F of the fluid can be derived from the acoustic energy density E [1,37]

(4) $$E=\frac{1}{2}\frac{p_{1}{}^{2}}{\rho_{0}c_{0}{}^{2}}+\frac{1}{2}\rho_{0}\vec{v_{1}}\cdot \vec{v_{1}}$$

(5) $$\vec{F}=-\langle \nabla E\rangle =-\nabla \langle E\rangle$$

where $p_{1}$ is the first-order acoustic pressure, and $v_{1}$ is the first-order acoustic velocity. Finally, the “Particle Tracing for Fluid Flow” physics model was used to obtain the trajectory of particles in this microfluidic device. To balance the simulation accuracy and the computational load, it is necessary to optimize the element size length of the computational mesh. Taking viscous boundary layer into consideration, the boundary layer thickness is $\delta ={\left(2v/\omega \right)}^{1/2}$ [38], where v is the kinematic viscosity of the medium $\nu =\eta_{0}/\rho_{0}$, $\eta_{0}$ is 0.893 mPa·s and δ=0.203 μm can be gotten. ω is the angular frequency of the wave. The maximum grid element size of this model is given by 10δ. The flow in the bulk wave is due to the effect of the boundary flow and the acoustic flow. Because the boundary layer thickness is small, the boundary flow has little effect on the particles in the fluid [39,40]. Thus, in this paper, it is only necessary to study only the role of acoustic flow. The laminar flow was also chosen to account for particle arrangement in COMSOL simulation. All other parameters were set in correspondence with the experiment.

### 4.1. One-Dimensional Patterning

**Figure 2:**
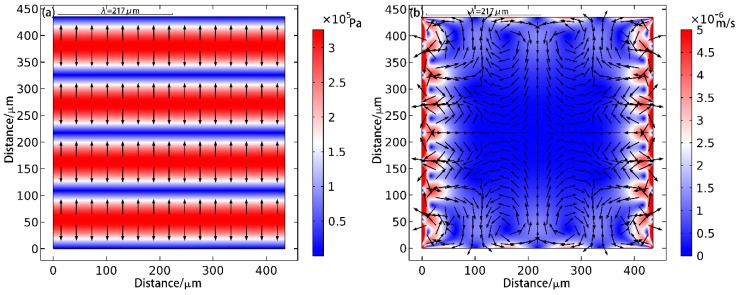
Simulation of one-dimensional particle patterning. In the figure (**a**), the background color represents the amplitude of the sound pressure, with blue for the node, red for the antinode, and the arrow indicating the direction of the sound radiation force. The figure (**b**) is the simulation result of microstreaming in the cavity, and the arrows represent the direction of the liquid flow and the background color represent the velocity of the microstreaming.

The simulation result for a one-dimension particle patterning is shown in Figure 2.

As shown in Figure 2a, the ARF points to the node positions as expected. From Figure 2b, it can be seen that the flow velocity of the media in the ranges adjacent to the boundaries is larger than the one at the center.

**Figure 3:**
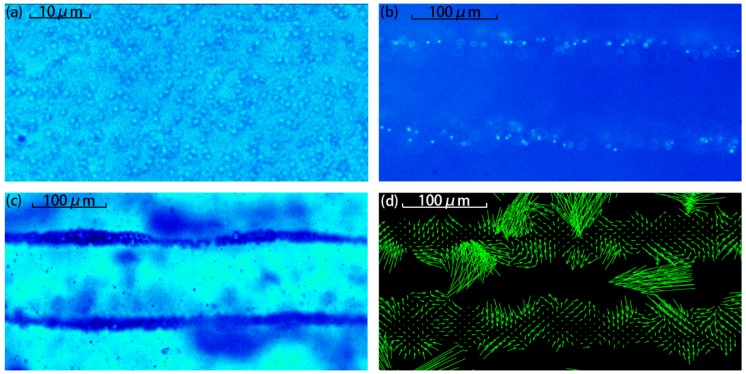
Experiment of one-dimension particle patterning (diameter = 1, 3, and 10 μm) is demonstrated in the (**a**–**c**) in sequence. The figure (**d**) is the 3μm particle velocity field calculated by PIV system.

In the experiment measurement, only one pair of piezoelectric plates was activated in phase. The applied peak to peak electric input voltage was 10 V. Figure 3a–c compare the patterns from the microspheres with *d* = 1, 3, and 10 μm. As shown, the ARF can pattern large microspheres (*d* = 3 μm and *d* = 10 μm) as strip shapes but not for small ones (*d* = 1 μm).

Figure 3d shows the velocity field from the 3 μm diameter particles using the PIV technique. As the image shows the later period of particle aggregation, few particles exist outside the aggregation area (the stripe). Too few particles leads to difficulties in the calculation of the PIV result, explaining the absence of arrows in some regions. As shown, most the velocity vectors point to the nodes. Though halfway between the two nodes, the force on the particle is zero in theory; it is not the stable equilibrium position of the particles. Even a little perturbation will cause the particles to move away from these positions to the node quickly. However, in the nodal area, the particles are so slowly gathered that the ARF is also very low. In addition, it can also be seen that there are spirals in the field and this indicates that the particles in the patterning process are not in a stable state. This is because the particles are also affected by the micro-fluid and the acoustic vortex, as well as some small perturbations in the sound field, which are also observed in the experiments.

### 4.2. Two-Dimensional Patterning

**Figure 4:**
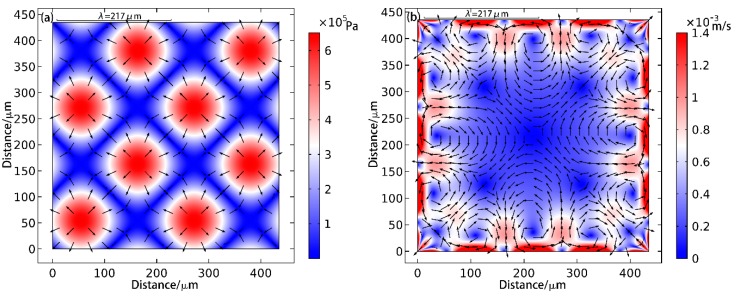
Simulation of two-dimensional particle patterning. In the figure (**a**), the background color represents the amplitude of the sound pressure, with blue for the node, red for the antinode, and the arrow indicating the direction of the sound radiation force. The figure (**b**) is the simulation result of microstreaming in the cavity, and the arrows represent the direction of the liquid flow and the background color represents the velocity of the microstreaming.

Figure 4 shows the simulation results for two-dimensional patterning. In Figure 4a, the nodal positions appear as inclined strips (termed as inclined strip patterns) and the positions of the antinodes are shown as a few dots (termed as lattice patterns). From Figure 4a it can be seen that, in the direction of wave propagation, the distance between the antinodes is a wavelength. As shown in Figure 4b, since four transducers are simultaneously emitting ultrasound waves in two-dimensions, the overall amplitude flow field is larger than that of the one-dimensional case. Again, flow field in the region adjacent to the boundary layers is higher than that at the center. The radiation distribution pattern is thus easy to understand. Meanwhile, the relevant mathematical derivation can be found in reference [9]. Through the sound pressure derivation in the standing wave field, we can acquire the stripe-like sound field distribution.

**Figure 5:**
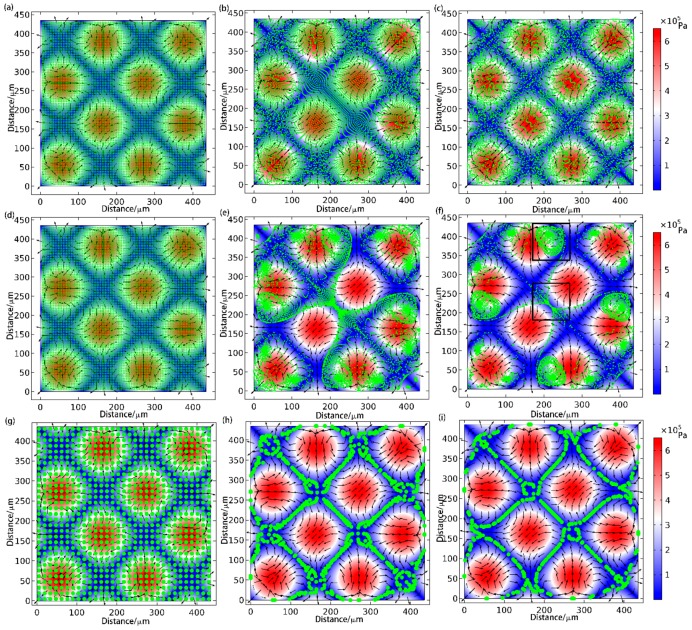
Simulation results in the continuous time with the arrows representing the flow velocity of the microfluidics, the color of the background the magnitude of the sound pressure in the standing wave field, and the green particles the polystyrene microspheres. (**a**–**i**) are the pattern of particles whose diameters are 1, 3, and 10 μm aggregation on different times. (To provide better visual effects, the 1 μm particles are zoomed in three times).

In order to investigate the effect of particle size on the AS force, the particle-tracking module in COMSOL^®^ software was used and the simulation results are shown in Figure 5. As shown in Figure 5a–c for the small size particles (d = 1 μm), they were not trapped as expected. For the medium size particles (d = 3 μm), as shown in Figure 5d–f, the particles first travelled along the nodal lines (pressure is low), then flowed along the acoustic streaming and finally clustered as a big dot (as the black box shown in Figure 5f, resulting in a lattice pattern in which the distance between two dots is around a wavelength along the direction of sound wave propagation. It is noted that the particles were finally trapped in some dots of the strip and generated a similar lattice pattern as the particle patterning under the condition of φ<0, as well as with a large lattice gap (around a wavelength). This pattern is generated by the combination functions of ARF and AS on the medium-size particles. For the large size particles (d = 10 μm), as shown in Figure 5g–i, the particles travelled along the nodal strips and finally formed an inclined strip pattern as expected, which is similar to the result in [9].

**Figure 6:**
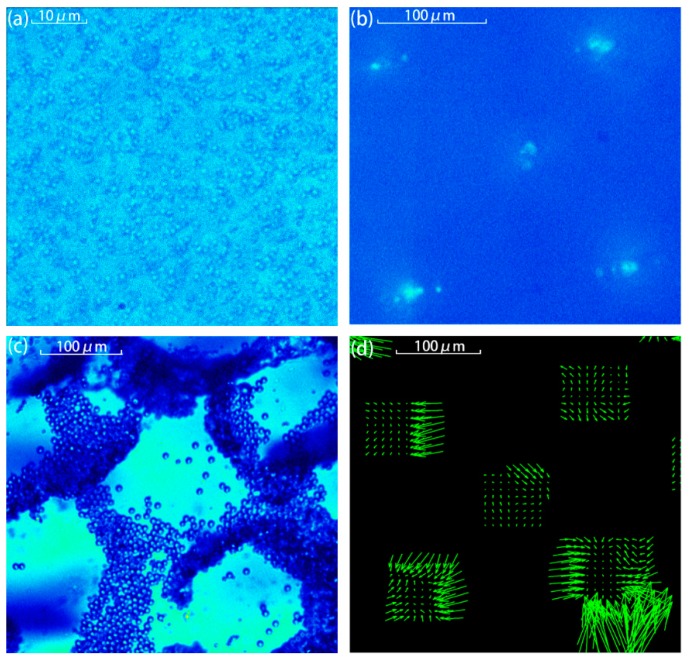
The two-dimension pattern of particles with a diameter of (**a**) 1 μm, (**b**) 3 μm, and (**c**) 10 μm. (**d**) is the velocity field from 3 μm particles measured by the PIV system.

In the experimental measurements, two pairs of piezoelectric plates were energized with peak-peak voltage of 10 V in phase. The experimentally measured results are shown in Figure 6. Comparing Figure 5 and Figure 6, it can be seen that the particle patterns agreed well, i.e., no pattern for 1 μm-diameter particles, lattice shape pattern with a lattice separation distance around a wavelength for 3 μm-diameter particles, and inclined strip shape pattern for 10 μm-diameter particles. Figure 6d shows the flow velocity field for 3 μm-diameter particles. As shown in Figure 6d, the whole flow field appeared in a lattice pattern, and the velocities of the particles points to the site where particles gathered. It can be also seen that particle velocity points to the gathering point. For the same reason as the 1D situation, it can be seen that some of the arrows do not point to the center of gathered area and that no arrows appear in some areas. It is noted that, from Figure 5d–f and Figure 6b, for the medium size particles, the final pattern is determined by the combination functions of the ARF and the AS, i.e., in the case of 3 μm-diameter polystyrene microspheres, they should be arranged in a stripe shape in two-dimensional acoustic field if only affected by the ARF, but they eventually clustered into a lattice due to the presence of microfluidics. This also indicates that the movement of particles of medium size are dominated by the influence of ARF, but the acoustic fluid effect also needs to be taken into consideration once particles have aggregated in the strip so that particles would be further pushed into the acoustic potential well where the velocity of acoustic fluid remains small. It should be explained that the gathered area is not the anti-node, it just appears in the same style as the anti-node. The simulation results also show that the area where the particles aggregated is where acoustic fluid is small (Figure 5f). This conjecture is confirmed in both simulations and experiments.

## 5. Conclusions

In this paper, particles with various sizes (d = 1 μm, 3 μm and 10 μm) were used in the acoustic standing wave fields to investigate the effect of ARF and AS on particle patterning. The PIV technique was used to observe the flow fields in acoustic standing wave fields in water and it helped to understand the force condition on particles. Through the simulation and PIV measurement techniques, it is shown that, in one-dimensional acoustical fields, ARF is dominant in patterning for medium and large size particles (d = 3 μm and 10 μm). In two-dimensional acoustical fields, ARF is still dominant in patterning for large size particles (d = 10 μm) while the combined effect of ARF and AS leads to the final pattern of medium size particles (d = 3 μm). The effect of AS plays a subject role in the motion of 1 μm diameter particles. According to [20], if the diameters of particles are larger than 2 μm, the ARF plays a subject role in the motion of the particles and the motion has a good agreement with the theory if the diameter is larger than 5 μm. This conclusion coincides with the conclusion of this paper, both in simulation and experiment. 1 μm particles under the domination of the AS cannot be arranged into a certain pattern; 10 μm particles which are ARF dominated can be arranged into a grid; 3 μm particles under combined effect of ARF and AS gathered into some big dots. The experiment and simulation is performed in the square standing wave area in the 6.9 MHz, but it has universality in the condition of plane standing waves because the force situation of the particles remains the same in this case [1]. Besides, the result is applicable for other frequencies. The change in frequency is equivalent to the change in the relative magnitude of the wavelength and particle size. As the motion of particles with multiple sizes has been researched in the article. The conclusion can thus be applied and modified for particles of other sizes and the acoustic wave of other frequencies. By considering the interaction between particles with various sizes and their surrounding ARF and AS, the findings will help in the design of acoustic-driven microfluidic devices to increase the diversity of particle patterning. This will also help to ensure the stability and repeatability of acoustofluidic devices and accelerate their industrial application.
